# Different remote realities: health and the use of territory in Brazilian rural municipalities

**DOI:** 10.11606/s1518-8787.2022056003914

**Published:** 2022-08-01

**Authors:** Aylene Bousquat, Márcia Cristina Rodrigues Fausto, Patty Fidelis de Almeida, Juliana Gagno Lima, Helena Seidl, Amandia Braga Lima Sousa, Ligia Giovanella

**Affiliations:** I Universidade de São Paulo Faculdade de Saúde Pública Departamento de Política, Gestão e Saúde São Paulo SP Brasil Universidade de São Paulo . Faculdade de Saúde Pública . Departamento de Política, Gestão e Saúde . São Paulo , SP , Brasil; II Fundação Oswaldo Cruz Escola Nacional de Saúde Pública Sérgio Arouca Escola de Governo em Saúde Rio de Janeiro RJ Brasil Fundação Oswaldo Cruz . Escola Nacional de Saúde Pública Sérgio Arouca . Escola de Governo em Saúde . Rio de Janeiro , RJ , Brasil; III Universidade Federal Fluminense Instituto de Saúde Coletiva Niterói RJ Brasil Universidade Federal Fluminense . Instituto de Saúde Coletiva . Niterói , RJ , Brasil; IV Universidade Federal do Oeste do Pará Instituto de Saúde Coletiva Santarém PA Brasil Universidade Federal do Oeste do Pará . Instituto de Saúde Coletiva . Santarém , PA , Brasil; V Universidade de São Paulo Faculdade de Saúde Pública Programa de Pós-Graduação São Paulo SP Brasil Universidade de São Paulo . Faculdade de Saúde Pública . Programa de Pós-Graduação . São Paulo , SP , Brasil; VI Fundação Oswaldo Cruz Instituto Leônidas e Maria Deane Manaus AM Brasil Fundação Oswaldo Cruz . Instituto Leônidas e Maria Deane . Manaus , AM , Brasil; VII Fundação Oswaldo Cruz Centro de Estudos Estratégicos Rio de Janeiro RJ Brasil Fundação Oswaldo Cruz . Centro de Estudos Estratégicos . Rio de Janeiro , RJ , Brasil

**Keywords:** Sociocultural Territory, Health policies, Rural Population Health, Health Care Models

## Abstract

**OBJECTIVE:**

To characterize remote rural Brazilian municipalities according to their logic of insertion into socio-spatial dynamics, discussing the implications of these characteristics for health policies.

**METHODS:**

Starting from the category of analysis – the use of the territory – a typology was elaborated, with the delimitation of six clusters. The clusters were compared using socioeconomic data and the distance in minutes to the metropolis, regional capital, and sub-regional center. Mean, standard error and standard deviation of the quantitative variables were calculated, and tests on mean differences were performed.

**RESULTS:**

The six clusters identified bring together 97.2% of remote rural municipalities and were called: “Matopiba,” “Norte de Minas,” “Vetor Centro-Oeste,” “Semiárido,” “Norte Águas,” and “Norte Estradas.” Differences are observed between the clusters in the analyzed variables, indicating the existence of different realities. Remote rural municipalities of “Norte Águas” and “Norte Estradas” clusters are the most populous, the most extensive and are thousands of kilometers away from urban centers, while those in “Norte de Minas” and “Semiárido” clusters have smaller areas with a distance of about 200 km away from urban centers. The remote rural municipalities of the “Vetor Centro-Oeste” cluster, in turn, are distinguished by a dynamic economy, inserted into the world economic circuit due to the agribusiness. The Family Health Strategy is the predominant model in the organization of primary health care.

**CONCLUSION:**

Remote rural municipalities are distinguished by their socio-spatial characteristics and insertion into the economic logic, demanding customized health policies. The strategy of building health regions, offering specialized regional services, tends to be more effective in remote rural municipalities closer to urban centers, as long as it is articulated with the health transportation policy. The use of information technology and expansion of the scope of telehealth activities is mandatory to face distances in such scenarios. Comprehensive primary health care with a strong cultural component is key to guaranteeing the right to health for citizens residing in such regions.

## INTRODUCTION

The provision of health services in rural areas that are far from urban centers remains a major challenge for health systems in the twenty-first century.

Constraints related to the access to services with a greater technological density; difficulties with transportation and communication, and lack of health professionals, especially physicians, among other problems, are exhaustively described in the literature ^1^ .

The Brazilian reality is no exception to this scenario; there is a concentration of health services in urban centers and in the most economically dynamic areas, and the population residing in rural areas not only faces greater difficulties in accessing health services, but also has worse life and health conditions ^4 , 5^ . In general, rural municipalities have higher percentages of low-income families, high rates of illiteracy, and a higher incidence of neglected diseases. Furthermore, their economies are fragile and dependent on central governments’ fund transfers.

Using the terminology proposed by Santos and Silveira ^6^ , these areas could be called “opaque territories,” as they would maintain more tenuous relationships with the global economic circuit, opposed to bright areas that maintain intense relationships. From the perspective of access to health, it can be said that these are areas in which the Inverse Care Law is still current, that is, the availability of health resources is scarcer where less privileged social groups with greater health needs reside ^7^ .

Although the differences in access to health services between rural and urban populations have diminished with the Unified Health System (SUS) implementation, inequalities are still glaring ^8^ . We recognize that inequities in access to health services are not restricted to the rural-urban binomial, but observed in the most different scenarios. However, this article focus on rural areas, especially those located far from urban centers. Undoubtedly, this theme is central to the formulation and planning of public policies, especially in a country with continental dimensions and marked by a pattern of extreme socio-spatial inequality such as Brazil.

In 2017, the *Instituto Brasileiro de Geografia e Estatística* (IBGE – Brazilian Institute of Geography and Statistics) ^9^ proposed a new characterization of the Brazilian territory, dividing the municipalities into urban, adjacent intermediate, remote intermediate, adjacent rural, and remote rural. Two elements were central to the classification: travel time to a regional sub-center, center or metropolis, and the population residing in densely occupied areas. Based on this classification, 323 municipalities were characterized as remote rural, with a total population of 3,856,692 Brazilian citizens.

In the previous paragraphs, traits common to Remote Rural Municipalities (RRMs) were detailed, but what sets them apart? Are population sparseness and distance from urban centers sufficient to characterize them, to design health policies? At the end of 2019, a new primary health care (PHC) financing policy, proposed by the Ministry of Health ^10^ , included the IBGE classification as one of the criteria for transferring resources to municipalities, without further reflection on the various aspects involved. There are common traits in RRMs; however, in order to better inform the formulation of policies, it is important to investigate their particularities.

Santos and Silveira ^6^ demonstrate that Brazilian socio-spatial development is marked by a very unequal insertion of different places into the economic circuit. We believe that the use of this theoretical framework can contribute to a better understanding of the reality/realities of RRMs and its/their effects on the health system configuration ^11^ . From this perspective, this article aims to characterize the RRMs according to their logic of insertion into the Brazilian socio-spatial dynamics, discussing the implications of these characteristics for health policies.

## METHODS

Starting from the category of analysis – the use of the territory – the RRMs typology was elaborated. ^11^ It was based on the study by Santos and Silveira ^6^ , who proposed a Brazilian regional division, in which “4 Brazils” are identified: the Concentrated Region (South and Southeast); the Region of Recent Peripheral Occupancy; the Northeast; and the Amazon. The RRMS were plotted on the map according to these “4 Brazils,” and the areas with the highest concentration of these municipalities were identified. Subsequently, the respective logics of insertion into the economic circuit and its main form of interconnection with the other points of the territory (land or river) were analyzed, based on the data available on the intermodal maps of the National Department of Infrastructure and Transportation and IBGE ^12^ , which included the main economic activities, dependence on government fund transfers, per capita Gross Domestic Product (GDP), population density, and the percentage of the population receiving *Bolsa Família* . These variables were chosen due to their importance in the analysis of access to health in remote rural settings ^13^ . In the case of *Bolsa Família* , the percentage of the covered population was calculated considering the national average of 3.4 people per family, with data obtained from the *Caixa Econômica Federal* website. The qualitative analysis led to the design of six clusters that bring together 313 RRMs, named: “Matopiba,” “Norte de Minas” [North of Minas Gerais state], “Vetor Centro-Oeste” [Central-West Vector], “Semiárido” [Semiarid Region], “Norte Águas” [North Waters] and “Norte Estradas” [North Roads].

Next, the distances from the metropolis, regional capital, and sub-regional center were calculated for each of the clusters, considering the time required to travel them. The distance and time variables were provided by the IBGE Geosciences Directorate. Municipalities were categorized as metropolis, regional capital or sub-regional center according to IBGE classification ^14^ . The main urban centers, large cities, and extensive area of direct influence are considered metropolises. Regional capitals, in turn, have management capacity at the level immediately below, with a smaller area of influence. The sub-regional centers are characterized by managing less complex activities.

Statistical analysis was performed using IBM-SPSS version 22 software, considering a 5% significance level. Mean, standard error, and standard deviation of the quantitative variables were calculated and tests of mean differences between the clusters were performed. The statistical test used for variables with normal distribution was the analysis of variance (ANOVA), and Kruskal-Wallis test for the others, followed by Bonferroni post-hoc.

To demonstrate the different health realities of the RRMs, some selected health indicators were calculated for each of the clusters and for the set of RRMs: inhab. consultations/year; inhab. visits by community health workers/year; coverage of the Family Health Strategy (ESF); SUS hospitalizations (100 inhab/year); SUS high complexity hospitalizations/(1,000 inhab/year); lives covered by private health insurance plans; infant mortality rate; percentage of deaths from ill-defined causes; percentage of hospitalizations for PHC-sensitive conditions; live births with adequate prenatal care, and percentage of patients who started cancer treatment more than 60 days after diagnosis. As a way of approaching the composition of the population, especially considering the aforementioned ‘invisibility’ of populations residing in remote rural areas, data were collected on the percentage of indigenous people in the population.

This analysis is part of the research “APS em territórios rurais e remotos no Brasil,” approved by the research ethics committee of the National School of Public Health Sérgio Arouca (ENSP) with statement No. 2.832.559.

## RESULTS

**Figure 1:**
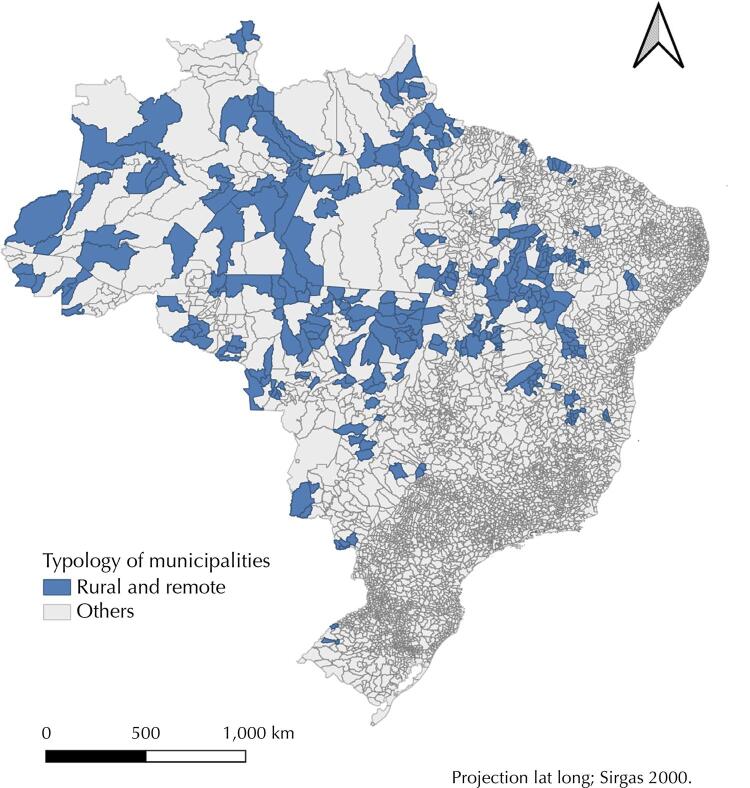
Brazilian remote rural municipalities, 2017.

Source: Elaborated from the IBGE classification (2017).

In Figure 1 , it is possible to visualize the distribution of RRMs in the Brazilian territory; there is a concentration of these municipalities in three of the “4 Brazils,” and the absence of these municipalities in the Concentrated Region is evident, with the exception of a grouping in the North of Minas Gerais state. The six clusters identified agglutinate 96.9% of the RRMs, and the main characteristics of the clusters can be seen in Table 1 .

**Table 1:** Socioeconomic characteristics of remote rural municipalities per clusters.

Clusters	n	Population ^a^		Área (km ^2^ ) ^b^		Density (inhab./km ^2^ ) ^c^		per capita GDP (R$) ^d,e^		Population with Bolsa Familia (%) ^f^	
		Mean	SD	Mean	SD	Mean	SD	Mean	SD	Mean	SD
1. Vetor Centro-Oeste	84	9,151.4	6,730.1	5,885.8	5,120.3	2.3	2.1	34,084.5	31,374.7	21.4	9.10
2. Norte Minas Gerais	22	9,271.7	5,820.7	1,059.3	897.8	11.26	5.6	7,475.3	816.4	45.6	9.07
3. Matopiba	92	8,321.3	7,806.0	2,652.0	2,399.1	4.47	4.0	11,860.8	7,394.9	50.3	15.0
4. Norte Estrada	28	20,703.6	13,465.2	13,284.8	12,776.6	2.80	3.9	12,791.0	4,998.7	48.3	16.3
5. Norte Água	45	21,002.1	4,532.0	14,997.1	17,155.2	3.32	3.3	8,539.1	3,158.0	54.9	11.8
6. Semiárido	42	11,706.6	11,420.5	1,847.0	2,411.0	10.06	8.7	6,626.8	870.2	64.0	8.95

GDP: Gross Domestic Product; SD: standard deviation.

^a^ Kruskal-Wallis p < 0.01. Post hoc multiple comparisons 1 ≠ 4,5; 2 ≠ 4,5; 3 ≠ 4,5; 4 ≠ 6; 5 ≠ 6.

^b^ Kruskal-Wallis p < 0.01. Post hoc multiple comparisons 1 ≠ 2,3,5,6; 2 ≠ 3,4,5; 3 ≠ 4,5; 4 ≠ 6; 5 ≠ 6.

^c^ Kruskal-Wallis p < 0.01. Post hoc multiple comparisons 1 ≠ 2,3,5,6; 2 ≠ 3,4,5; 3 ≠ 4,5; 4 ≠ 6; 5 ≠ 6.

^d^ Kruskal-Wallis p < 0.01. Post hoc multiple comparisons 1 ≠ 2,3,4,5,6; 2 ≠ 4; 3 ≠ 5,6; 4 ≠ 5,6.

^e^ Calculation from IBGE data for the year 2018.

^f^ Data obtained from the *Caixa Econômica Federal* website, considering the national average of 3.4 people per family.

The cluster in the North of Minas Gerais State can be considered the expression of one of the “opaque zones” of the concentrated region, considerably mirroring the uneven process of constitution of the Minas Gerais state’s territory. Unlike other Minas Gerais regions, it is characterized by a low insertion into the economic circuit, with great socioeconomic needs, although it is an area of ancient occupation of the Brazilian territory.

In the Northeast, two clusters were identified, “Semiárido” and “Matopiba,” with different insertions into the national economy. “Matopiba,” an acronym for the initials of Maranhão, Tocantins, Piauí and Bahia states, is a cerrado biome area, recently delimited by the *Instituto Nacional de Colonização e Reforma Agrária* (Incra – National Institute for Colonization and Agrarian Reform) and the *Empresa Brasileira de Pesquisa Agropecuária* (Embrapa – Brazilian Agricultural Research Corporation). One of the main features of occupation of this territory lies in the recent changes in land use and land tenure, which include the introduction of new production technologies and the expansion of agribusiness. This new agricultural frontier collides with the existence of thousands of people who already lived there, making traditional use of the cerrado ^15^ .

In turn, “Semiárido,” an area of ancient occupation of the Brazilian territory and with low insertion into the economic circuit, is a region marked by drought, with impacts on economic, social, and environmental development that are reflected in worse social and health indicators. Although it is a climatic event, its impact depends on human activities, social vulnerability, and public policy responses ^16^ .

In “Vetor Centro-Oeste,” the technical-scientific informational environment was established on a territory of rarefied technical heritage, which absorbed the renewal through modern agricultural production associated with livestock with corporate use of the territory. The not only technological but also ideological aspect that sustains it can be described in globalized agriculture, combined with the participation of the State via financing. Certainly it is the cluster that is most integrated into the global economic circuit, through agribusiness ^6^ , adding 84 RRMs, including those from Rondônia, which are functionally integrated into this vector as a result of the expansion of soybean and agriculture.

The North Region is characterized by demographic rarefactions and low technical density, inherited from past periods of the Brazilian spatial occupation process. The vastness of the territory influences the configuration of interconnection points; where river points were and are central. It is a forest biome territory with a vast complexity of ecological and social relationships ^17^ , and its history is marked by outbreaks of external intervention and predatory exploitation of natural resources. The South and East of the region are zones of tension over land disputes with agribusiness, in which the expansion of capitalism took place simultaneously with the creation of companies under the State encouragement direction, with a marked emphasis on the road transportation matrix ^18^ .

From these different logics, two clusters were delimited: “Norte Águas” and “Norte Estradas.” The former agglutinates the RRMS marked by the dynamics of the rivers, the latter those guided by the highways. A mixed logic was observed in seven municipalities; in the past their dynamics were guided by the rivers, but highways have been built recently, and because of this change it was decided to consider them as members of the “Norte Estradas” cluster. In “Norte Águas,” the complexity of the dynamics of the rivers impacts the population’s entire life. The river is the means of access to water, transportation, leisure, and any type of services. At the low tide time of the river, some communities are completely isolated due to the difficulty of access by water ^19^ . On the other hand, in “Norte Estradas,” the main landmarks are the highways, such as the Transamazônica and Santarém-Cuiabá. While the RRMs have a percentage of indigenous people almost 10 times higher than the whole Brazilian population, these two clusters have even higher percentages, reaching more than 9% in “Norte Águas” ( Table 2 ).

**Table 2:** Health and demographics indicators selected. Remote rural municipalities and clusters, Brazil.

Indicator	Brazil	RRMs	Clusters					
			Vetor Centro-Oeste	Norte de Minas	Matopiba	Norte Estrada	Norte Água	Semiárido
Inhab. consultations/year ^a^	1.79	1.65	2.85	3.07	1.20	0.87	0.95	2.16
Inhab. visits by ACSs /year ^e^	1.57	2.58	2.67	2.93	2.31	2.19	2.81	2.70
Coverage of the ESF (%) ^g^	63.7	85.78	83.50	100	98.04	73.76	78.19	91.35
SUS hospitalizations (100 inhab./year) ^a^	5.88	5.58	6.14	6.27	6.27	5.72	4.45	5.00
SUS high complexity hospitalizations/ (1,000 inhab./year) ^a^	4.4	1.3	2.0	3.1	1.4	0.7	0.6	1.6
Lives covered by private health insurance plans (%) ^c^	22.4	1.50	3.97	0.86	1.16	0.85	0.33	0.72
Infant mortality rate ^a^	12.39	17.56	7.10	16.95	16.56	20.79	17.00	16.55
Deaths from ill-defined causes (%) ^a^	6.06	10.94	8.93	15.23	9.25	13.15	13.92	9.37
Hospitalizations for PHC-sensitive conditions (%) ^d^	30.6	41.14	33.19	38.28	42.77	42.98	41.27	48.36
Live births with adequate prenatal care (%) ^a,b^	70.76	52.81	66.82	71.97	58.9	45.72	39.85	62.3
Patients who started cancer treatment in the SUS more than 60 days after diagnosis (%) ^a^	17.7	21.7	18.3	27.4	21.8	24.5	23.2	27
Indigenous people in the population (%) ^f^	0.43	4.62	4.42	4.14	1.99	6.17	9.17	0.22

ACS: community health worker; ESF: family health strategy; RRM: remote rural municipalities; SUS: Unified Health System; PHC: primary health care.

^a^ Calculated from data available on Datasus, year 2019 reference.

^b^ Start of prenatal care in the first trimester and at least six prenatal consultations.

^c^ Calculated from data available from the National Health Agency, June 2019 reference.

^d^ Data available on Datasus, reference year 2015.

^e^ Calculated from data available on the Health Information System for Primary Care, year 2019 reference.

^f^ 2010 Census data.

^g^ Calculated from data available on e-Gestor Primary Care, June 2019 reference.

Most RRMs have their Municipal Human Development Index (MHDI) classified as low; only 13 are in the highest range, and 12 of which are in the “Vetor Centro-Oeste.” “Norte Águas,” in turn, has the highest concentration of municipalities with IDHM in the “Very Low” range. The fragile economies of the RRMs can be evidenced by the important weight of the public administration in the local economies, with 81.2% having the public service as the main activity that adds value to the economy. The exceptions are fundamentally located in “Vetor Centro-Oeste” and in “Matopiba.” In “Vetor Centro-Oeste,” municipalities that have agriculture as their main activity and livestock as a secondary activity stand out, and in “Matopiba” agriculture is the main occupation.

Differences are observed between the clusters with regard to the number of inhabitants, area, population density, per capita GDP, and percentage of the beneficiary population of the *Bolsa Família* (brazilian income transfer program) ( Table 1 ). The analysis of per capita GDP shows in a clear manner the important insertion into the economic circuit of “Vetor Centro-Oeste” RRMs and, to a lesser extent, of Matopiba RRMs, while the *Bolsa Família* coverage follows the opposite pattern.

**Figure 2:**
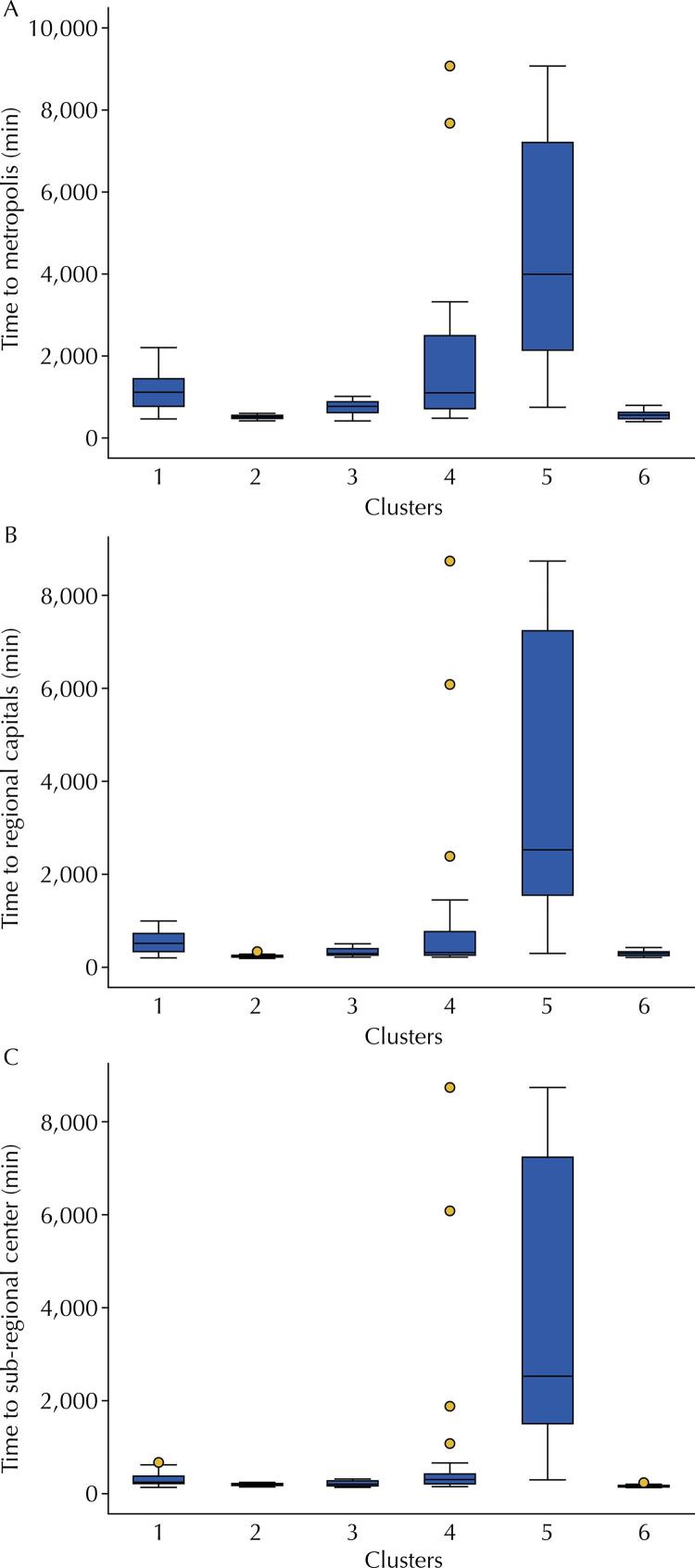
Travel time between remote rural municipalities and metropolises, regional capitals, and the sub-regional center.

Another central point is the distance of the RRMs from the municipalities classified within the scope of higher urban hierarchies. This data can be understood as an indirect measure of the difficulty that the population faces in reaching urban centers, where medium and high complexity outpatient procedures and consultations and hospitalizations are often offered. Figure 2 shows the times in minutes to cover the distances from the six clusters to the metropolis, regional capital, or a sub-regional center. Differences are observed (p < 0.01) in all cases, indicating the existence of different remote realities and not just a single one. “Norte Águas” has the greatest distances between its municipalities and the hierarchically higher urban centers, with an average of 4,565 minutes to the metropolis. For 14 RRMs, the connection is made directly with the metropolises, especially Manaus, without an intermediate urban network, and in 10 it is directly with the regional capitals.

A. Kruskal-Wallis p < 0,01. Post hoc multiple comparisons 1 ≠ 2, 3, 5, 6; 2 ≠ 3, 4, 5; 3 ≠ 4, 5 , 6; 4 ≠ 5, 6 ; 5 ≠ 6.

B. Kruskal-Wallis p < 0,01. Post hoc multiple comparisons 1 ≠ 2, 3, 5, 6; 2 ≠ 3, 4, 5; 3 ≠ 5; 4 ≠ 5; 5 ≠ 6.

C. Kruskal-Wallis p < 0,01. Post hoc multiple comparisons 1 ≠ 2, 5, 6; 2 ≠ 4, 5; 3 ≠ 4, 5, 6; 4 ≠ 5, 6; 5 ≠ 6.

The municipalities of “Norte Estradas” also face great distances; a little less than half of the municipalities (13) connect directly to the regional capitals and the metropolises Manaus and Belém. The sub-regional centers reflect considerably the process of occupation and growth of these municipalities, settled by roads, such as Altamira, place of environmental conflicts and land occupation during the construction of the Belo Monte hydroelectric plant.

“Vetor Centro-Oeste” RRMs present distances around 1,000 minutes to the metropolis and 500 minutes to the regional capitals. If these times seem short compared to the time of the clusters mentioned above, they are still very relevant. Only eight RRMs in this cluster are directly related to the metropolis or regional capital, while 62 establish a relationship with zone and sub-region centers, indicating the presence of a well-distributed network of cities.

“Matopiba” RRMs present average distances covered in about 750, 290, and 200 minutes for the metropolis, regional capital, and sub-regional center, respectively. As in “Vetor Centro-Oeste,” there is a wide connection with cities classified as zone and sub-region centers.

In the opposite direction, with much shorter distances and times and multiple interconnections with several hierarchically superior cities, there are “Norte de Minas” and “Semiárido.” In the former, regional capitals are 215 minutes apart, on average. In the latter, there are more regional capitals and metropolises, confirming the antiquity of the territory occupation processes.

In Table 2 , it is possible to observe important differences in the selected indicators of health and performance of health services, between both the set of RRMs and the rest of the country and between the clusters. As for the supply and organization of health services, the Family Health Strategy model predominates, with population coverage and number of visits by community health workers above the national average. Despite the high coverage, the issue of quality is a problem, as demonstrated by the lower percentage of live births with adequate prenatal care in all clusters, compared to the national scenario, except in the North of Minas Gerais state and in the behavior of Hospitalizations for PHC-sensitive conditions (ICSAPS). A strong and robust PHC demands an articulation with the other levels of the system, and the difficulties for this articulation are evident in the indicators of high complexity hospitalizations and percentage of patients who started cancer treatment more than 60 days after diagnosis. Expressing the low economic capacity of the population, private health insurance plans are practically non-existent ( Table 2 ).

## DISCUSSION

The absence of theoretical frameworks is an important gap in studies on health in remote rural settings ^20^ , and the incorporation of the theoretical framework of Santos and Silveira ^6^ allowed a better approach to the various Brazilian remote rural scenarios. The results found indicate the existence of different realities that can and should be considered from the perspective of health policies.

Internationally, policies aimed at guaranteeing access to health in rural areas have pointed to the need for: a health care model based on robust PHC, with a strong community and cultural component; broad telehealth initiatives; reinforcement of the logistics capacity to transport users and supplies, with different logics for specialized care, hospitalizations and emergency situations, and the introduction of strong mechanisms for retaining professionals in these regions ^21 , 22^ .

In the Brazilian case, it is imperative to formulate policies that dialogue with the particularities of the identified scenarios and reverse the situation observed in the health indicators presented. For this discussion, we take two central themes for Brazilian health policies as a reference: the construction of health regions and the care model. One of the first questions to be faced is: what is the regional response capacity in relation to the different insertions into the network of cities? The lack of a network of nearby cities, that is, connections of these RRMs with distant regional capitals or metropolises, certainly has a negative impact on the processes of building health regions. Undoubtedly, this design is more feasible in RRMs located at shorter distances from regional capitals and sub-centers, as it is the case of those in the Semiarid region and Northern Minas Gerais, where logistical investment in road transportation systems for users is mandatory, and experiences such as regional polyclinics can correct deficiencies in access to specialized care, as long as health transportation is guaranteed ^23^ . However, this same strategy becomes difficult to implement in those RRMs in which the population has to face immense displacements to hierarchically higher urban centers, as it is the case of the “Norte Água” and “Norte Estrada” clusters, where the local supply , closer to specialized services, even without economies of scale, is central to guaranteeing access for citizens residing in these regions. These needs have to be reflected in funding, especially at the state and national levels, as most RRMs have very fragile economies, unable to bear alone the high costs resulting from these strategies.

The lack of transportation is one of the most reported factors as a barrier for residents of rural areas to access more complex health services. The costs to carry out the displacement are often assumed directly by the users, compromising the meager family budget and configuring a catastrophic expense ^24^ . This service should be implemented, maintained and paid for by the three federated entities, in a solidary way. In Brazil, since 1999, there has been a payment forecast for a portion of these displacements through “Tratamento Fora de Domicílio” [Out-of-Home Treatment], but there are numerous weaknesses in this policy ^25^ .

Ensuring urgent care is a major challenge in scenarios such as these and reinstates the discussion of the role of small hospitals and mixed units. If, on the one hand, the literature points out constraints to the provision of quality care, on the other, the guarantee of first emergency care is mandatory, given the long travel times and distances involved. ^24^ The scope of health professionals‘ practices has to be expanded, with telehealth support, associated with a specific funding policy.

As for the care model, the data indicate an expressive presence of the Family Health Strategy in these municipalities, which is a great potential. The literature is fruitful in demonstrating that health care models based on PHC are associated with better performance of the health system in general. A strong PHC would be able to reduce the differences in health access and outcomes between rural and urban populations. However, the challenges faced in rural locations are even greater, demanding new designs for the provision of services, an expanded clinical competence, a strong community and cultural component, in addition to highlighting actions for promotion, prevention, and social participation. ^26^ Another positive point in the predominant model is the presence of community health workers, who are often the only guarantee of connecting a part of the population to health services; however, there is no preparation nor a planned follow-up so that they can act more efficiently in the face of this reality, which should be reviewed.

The most common types of PHC service provision for these regions are visiting models, in which teams travel from headquarters and go to more remote locations sporadically, even if at regular intervals, which makes timely treatment and continuity of care difficult. This strategy is based on the premise of the impossibility of retaining health professionals, especially physicians and nurses. Policies for retaining these professionals are certainly essential and the most successful ones have been articulated with the training processes since the undergraduate program. In Brazil, the *Mais Médicos* Program was an important initiative to reverse those care gaps ^27^ . Another positive point to be highlighted in the Brazilian primary care policy was the incorporation of the cultural dimension, well expressed in the *Política Nacional de Saúde Integral das Populações do Campo e da Floresta* (National Policy for the Comprehensive Health of Rural and Forest Populations).

Nevertheless, despite these positive aspects, much remains to be done. A central initiative for improving access to healthcare is the use of information technology. The most varied telehealth initiatives have been reported as important alternatives in the most diverse rural or remote scenarios worldwide ^28^ . The results found here, expressed in the immense distances between municipalities and urban centers, especially in “Norte Água,” “Norte Estrada” and “Vetor Centro-Oeste” clusters, suggest that information technologies are an essential investment to guarantee the reduction of inequities in access to health. The range of possibilities found in international experiences is very wide, especially in high-income countries that face this reality, going far beyond what has been proposed in Brazilian telehealth, including even examinations, procedures and urgency care, which are carried out jointly between local and remote teams ^29^ .

## CONCLUSIONS

This study shows that remote rural municipalities in Brazil are not homogeneous and that the different socio-spatial characteristics and insertion in the economic logic demand customized health policies for different realities. More solidary financing policies, adequacy of the designs of regional health networks, specific policies for the provision of human resources, in addition to an incentive for a robust PHC, with an expanded scope of practices and with a strong cultural and community component should be considered as priority policies by managers. The understanding of these particularities and the elaboration of specific policies for these territories are mandatory to guarantee the right to health for the citizens who reside there, with equity and integrality, contributing to make visible this often invisible portion of the Brazilian population.
