# Supplementary material for: Different remote realities: health and the use of territory in Brazilian rural municipalities
Source: Rev Saude Publica. 2022 Aug 1;56:73. doi: 10.11606/s1518-8787.2022056003914 (PMC9388062; doi:10.11606/s1518-8787.2022056003914)
Supplement: Translation

# Translation: Remoto ou remotos: a saúde e o uso do território nos municípios rurais brasileiros

**Authors:** Aylene Bousquat, Márcia Cristina Rodrigues Fausto, Patty Fidelis de Almeida, Juliana Gagno Lima, Helena Seidl, Amandia Braga Lima Sousa, Ligia Giovanella

**Affiliations:** I Brasil Universidade de São Paulo. Faculdade de Saúde Pública. Departamento de Política, Gestão e Saúde. São Paulo, SP, Brasil; II Brasil Fundação Oswaldo Cruz. Escola Nacional de Saúde Pública Sérgio Arouca. Escola de Governo em Saúde. Rio de Janeiro, RJ, Brasil; III Brasil Universidade Federal Fluminense. Instituto de Saúde Coletiva. Niterói, RJ, Brasil; IV Brasil Universidade Federal do Oeste do Pará. Instituto de Saúde Coletiva. Santarém, PA, Brasil; V Brasil Universidade de São Paulo. Faculdade de Saúde Pública. Programa de Pós-Graduação. São Paulo, SP, Brasil; VI Brasil Fundação Oswaldo Cruz. Instituto Leônidas e Maria Deane. Manaus, AM, Brasil; VII Brasil Fundação Oswaldo Cruz. Centro de Estudos Estratégicos. Rio de Janeiro, RJ, Brasil

**Keywords:** Território Sociocultural, Políticas de Saúde, Saúde da População Rural, Modelos de Assistência à Saúde

## Abstract

**OBJETIVO:**

Caracterizar os municípios rurais remotos brasileiros segundo suas lógicas de inserção na dinâmica socioespacial, discutindo as implicações dessas características para as políticas de saúde.

**MÉTODOS:**

Partindo da categoria de análise – o uso do território – elaborou-se uma tipologia, com delimitação de seis *clusters* . Os *clusters* foram comparados a partir de dados socioeconômicos e da distância em minutos para a metrópole, capital regional e centro sub-regional. Foram calculados a média, o erro padrão e o desvio padrão das variáveis quantitativas e realizados testes de diferenças de média.

**RESULTADOS:**

Os seis *clusters* identificados aglutinam 97,2% dos municípios rurais remotos e foram denominados de: Matopiba; Norte de Minas; vetor Centro-Oeste; Semiárido; Norte Águas; e Norte Estradas. Observam-se diferenças entre os *clusters* nas variáveis analisadas, indicando a existência de distintas realidades. Os municípios rurais remotos dos *clusters* Norte Água e Norte Estrada são os mais populosos, mais extensos e distam milhares de quilômetros de centros urbanos, enquanto os do Norte de Minas e do Semiárido tem áreas menores com distância de cerca de 200 km. Por outro lado, os municípios rurais remotos do vetor Centro-Oeste se diferem por uma economia dinâmica, inserida no circuito econômico mundial devido à presença do agronegócio. A Estratégia de Saúde da Família é o modelo predominante na organização da atenção primária à saúde.

**CONCLUSÃO:**

Os municípios rurais remotos distinguem-se em suas características socioespaciais e de inserção na lógica econômica, demandando políticas de saúde customizadas. A estratégia de construção das regiões de saúde, com oferta de serviços regionais especializados, tende a ser mais efetiva nos municípios rurais remotos mais próximos de centros urbanos, desde que articulada à política de transporte sanitário. O uso de tecnologia de informação e ampliação do escopo das atividades de telessaúde é mandatório para enfrentamento das distâncias em cenários como esse. A atenção primária à saúde integral com forte componente cultural é peça-chave para garantir o direito à saúde para os cidadãos que aí residem.

## INTRODUÇÃO

A oferta de serviços de saúde em áreas rurais e distantes dos centros urbanos persiste como um grande desafio para os sistemas de saúde no século XXI. Constrangimentos no acesso a serviços com maior densidade tecnológica; dificuldades com transporte e comunicação; escassez de profissionais de saúde, especialmente médicos, dentre outros problemas, são descritos exaustivamente na literatura ^1^ .

A realidade brasileira não foge a esse cenário, observa-se concentração dos serviços de saúde nos centros urbanos e nas áreas economicamente mais dinâmicas e a população que reside em áreas rurais não só enfrenta maiores dificuldades para acessar os serviços de saúde, como apresenta piores condições de vida e de saúde ^4 , 5^ . Em geral, os municípios rurais apresentam maiores percentuais de famílias de baixa renda, altas taxas de analfabetismo e maior incidência de doenças negligenciadas. Ademais, suas economias são frágeis e dependentes das transferências dos governos centrais.

Empregando terminologia proposta por Santos e Silveira ^6^ , essas áreas poderiam ser chamadas de territórios opacos, pois manteriam relações mais tênues com o circuito econômico global, em contraposição às áreas luminosas que mantêm relações intensas. Da perspectiva do acesso à saúde, pode-se afirmar que são áreas em que a *Inverse Care Law* é ainda atual, ou seja, a disponibilidade dos recursos em saúde é mais escassa onde residem os grupos sociais menos privilegiados e consequentemente com maiores necessidades em saúde ^7^ .

Embora as diferenças no acesso aos serviços de saúde entre as populações rurais e urbanas tenham diminuído com a implementação do SUS, as desigualdades ainda são gritantes ^8^ . Reconhecemos que as inequidades no acesso aos serviços de saúde não se restringem ao binômio rural-urbano, sendo observadas nos mais diferentes cenários. No entanto, o foco deste artigo são as áreas rurais, especialmente aquelas localizadas distantes dos centros urbanos. Sem dúvida, esse tema é central para a formulação e planejamento de políticas públicas, especialmente em um país com dimensões continentais e marcado por um padrão de extrema desigualdade socioespacial como o Brasil.

Em 2017, o Instituto Brasileiro de Geografia e Estatística (IBGE) ^9^ propôs uma nova caracterização do território brasileiro, dividindo os municípios em urbanos, intermediários adjacentes, intermediários remotos, rurais adjacentes e rurais remotos. Dois elementos foram centrais na classificação: o tempo de deslocamento até um subcentro, centro ou metrópole regional; e a população residente em áreas de ocupação densa. A partir dessa classificação, 323 municípios foram caracterizados como rurais e remotos , nos quais viviam 3.856.692 cidadãos brasileiros.

Nos parágrafos anteriores foram detalhados traços comuns aos municípios rurais remotos (MRR), mas o que os diferencia? Rarefação da população e distância de centros urbanos são suficientes para caracterizá-los, para elaborar políticas de saúde? No final de 2019, uma nova política de financiamento da atenção primária em saúde (APS), proposta pelo Ministério da Saúde ^10^ , incluiu a classificação do IBGE como um dos critérios de repasse de recursos para os municípios, sem maiores reflexões sobre os diversos aspectos aí envolvidos. Observam-se traços comuns aos MRR, contudo para melhor informar a formulação de políticas interessa investigar suas especificidades.

Santos e Silveira ^6^ demonstram que o desenvolvimento socioespacial brasileiro é marcado por uma inserção bastante desigual dos diversos lugares no circuito econômico. Acreditamos que a utilização deste referencial teórico pode contribuir para um melhor entendimento da(s) realidade(s) dos MRR e seus reflexos na configuração do sistema de saúde ^11^ . Dessa perspectiva, o presente artigo tem como objetivo caracterizar os MRR segundo suas lógicas de inserção na dinâmica socioespacial brasileira, discutindo as implicações dessas características para as políticas de saúde.

## MÉTODOS

Partindo da categoria de análise – o uso do território – elaborou-se proposta de tipologia dos MRR ^11^ . Tomou-se por base o estudo de Santos e Silveira ^6^ que propôs uma divisão regional brasileira, na qual são identificados “4 Brasis”: a Região Concentrada (Sul e Sudeste); a Região de Ocupação Periférica Recente; o Nordeste; e a Amazônia. Os MRR foram plotados no mapa conforme esses “4 Brasis”, identificando-se as áreas com maior concentração desses municípios. Na sequência, foram analisadas as respectivas lógicas de inserção no circuito econômico e sua principal forma de interligação com os demais pontos do territórios (terrestre ou fluvial), a partir dos dados disponíveis nos mapas intermodais do Departamento Nacional de Infraestrutura e Transportes e do IBGE ^12^ , que incluíram as principais atividades econômicas, a dependência das transferencias governamentais, o Produto Interno Bruto (PIB *) per capita,* a densidade populacional e o percentual da população que recebe Bolsa Família. Essas variáveis foram escolhidas pela sua importância na análise do acesso à saúde nos cenários rurais e remotos ^13^ . No caso do Bolsa Família, o percentual da população coberta foi calculado considerando a média nacional de 3,4 pessoas por família e os dados foram obtidos no site da Caixa Econômica Federal. A análise qualitativa levou ao desenho de seis *clusters* que aglutinam 313 MRR, denominados de: Matopiba; Norte de Minas; vetor Centro-Oeste; Semiárido; Norte Águas; e Norte Estradas.

A seguir, as distâncias da metrópole, capital regional e centro sub-regional foram calculadas para cada um dos *clusters* , considerando o tempo necessário para percorrê-las. As variáveis das distâncias e tempos foram cedidas pela Diretoria de Geociências do IBGE. A classificação das cidades como metrópole, capital regional ou centro sub-regional utilizada foi a do IBGE ^14^ . São consideradas metrópoles, os principais centros urbanos, cidades de grande porte e extensa área de influência direta. As capitais regionais, por outro lado, têm capacidade de gestão no nível imediatamente inferior, com menor área de influência. Já os centros sub-regionais se caracterizam por realizar gestão de atividades menos complexas.

A análise estatística foi realizada pelos softwares IBM-SPSS versão 22, considerando um nível de significância de 5%. Foram calculados a média, o erro padrão e o desvio padrão das variáveis quantitativas e realizados testes de diferenças de média entre os *clusters* . Nas variáveis com distribuição normal o teste estatístico foi a análise de variância (ANOVA) e nas demais Kruskal-Wallis, seguido do post-hoc de Bonferroni.

Para demonstrar as distintas realidades de saúde dos MRR foram calculados para cada um dos *clusters* e para o conjunto dos MRR alguns indicadores de saúde selecionados: consultas hab./ano; visitas de agentes comunitários de saúde hab./ano; cobertura da Estratégia da Saúde da Família; internações SUS 100 hab./ano; internações SUS de alta complexidade 1.000 hab./ano; vidas cobertas por planos de saúde privado; coeficiente de mortalidade infantil; percentual de óbitos por causas mal definidas; percentual de internações por condições sensíveis à APS; nascidos vivos com pré-natal adequado; e percentual de pacientes que iniciaram o tratamento de câncer com mais de 60 dias do diagnóstico. Como forma de se aproximar da composição da população, especialmente considerando a já referida ‘invisibilidade” das populações que residem em área rurais e remotas, foram coletados os dados sobre o percentual de indígenas na população.

Esta análise faz parte da pesquisa “APS em territórios rurais e remotos no Brasil”, aprovada no comitê de ética em pesquisa da Escola Nacional de Saúde Pública Sérgio Arouca (ENSP) com parecer nº 2.832.559.

## RESULTADOS

Na Figura 1 é possível visualizar a distribuição dos MRR no território brasileiro, observa-se concentração desses municípios em três dos “4 Brasis”, fica evidente a ausência desses municípios na Região Concentrada, com exceção de um grupamento no Norte de Minas Gerais.

**Figura 1:** Municípios rurais remotos brasileiros, 2017.

Fonte: elaborado a partir da classificação do IBGE (2017)

Os seis *clusters* identificados aglutinam 96,9% dos MRR, as principais características dos *clusters* podem ser visualizadas na Tabela 1 .

**Tabela 1:** Características socioeconômicas dos municípios rurais remotos por *clusters* .

Clusters	n	Habitantes ^a^		Área (km ^2^ ) ^b^		Densidade (hab/km ^2^ ) ^c^		PIB per capita (R$) ^d,e^		População com Bolsa Família (%) ^f^	
		Média	DP	Média	DP	Média	DP	Média	DP	Média	DP
1. Vetor Centro-Oeste	84	9.151,4	6.730,1	5.885,8	5.120,3	2,3	2,1	34.084,5	31.374,7	21,4	9,10
2. Norte Minas Gerais	22	9.271,7	5.820,7	1.059,3	897,8	11,26	5,6	7.475,3	816,4	45,6	9,07
3. Matopiba	92	8.321,3	7.806,0	2.652,0	2.399,1	4,47	4,0	11.860,8	7.394,9	50,3	15,0
4. Norte Estrada	28	20.703,6	13.465,2	13.284,8	12.776,6	2,80	3,9	12.791,0	4.998,7	48,3	16,3
5. Norte Água	45	21.002,1	4.532,0	14.997,1	17.155,2	3,32	3,3	8.539,1	3.158,0	54,9	11,8
6. Semiárido	42	11.706,6	11.420,5	1.847,0	2.411,0	10,06	8,7	6.626,8	870,2	64,0	8,95

PIB: Produto Interno Bruto; DP: desvio padrão.

^a^ Kruskal-Wallis p < 0,01. Pós-hoc comparações múltiplas 1 ≠ 4,5; 2 ≠ 4,5; 3 ≠ 4,5; 4 ≠ 6; 5 ≠ 6.

^b^ Kruskal-Wallis p < 0,01. Pós-hoc comparações múltiplas 1 ≠ 2,3,5,6; 2 ≠ 3,4,5; 3 ≠ 4,5; 4 ≠ 6; 5 ≠ 6.

^c^ Kruskal-Wallis p < 0,01. Pós-hoc comparações múltiplas 1 ≠ 2,3,5,6; 2 ≠ 3,4,5; 3 ≠ 4,5; 4 ≠ 6; 5 ≠ 6.

^d^ Kruskal-Wallis p < 0,01. Pós-hoc comparações múltiplas 1 ≠ 2,3,4,5,6; 2 ≠ 4; 3 ≠ 5,6; 4 ≠ 5,6.

^e^ Cálculo a partir de dados do IBGE para o ano de 2018.

^f^ Dados obtidos no site da Caixa Econômica Federal, considerando a média nacional de 3,4 pessoas por família.

O *cluster* do Norte de Minas pode ser considerado a expressão de uma das “zonas opacas” da região concentrada, espelhando sobremaneira o desigual processo de constituição do território mineiro. Diferentemente de outras regiões mineiras, se caracteriza por uma baixa inserção no circuito econômico, com grandes carências socioeconômicas, embora seja uma área de ocupação antiga do território brasileiro.

No Nordeste, dois *clusters* foram identificados, o do Semiárido e o de Matopiba, com inserções distintas na economia nacional. Matopiba, acrônimo das iniciais dos estados de Maranhão, Tocantins, Piauí e Bahia é uma área do bioma cerrado, recentemente delimitada pelo Instituto Nacional de Colonização e Reforma Agrária (Incra) e pela Empresa Brasileira de Pesquisa Agropecuária (Embrapa). Um dos principais traços de ocupação desse território reside nas mudanças recentes no uso e condição fundiária das terras, que inclui a introdução de novas tecnologias de produção e a expansão do agronegócio. Essa nova fronteira agrícola colide com a existência de milhares de pessoas que lá já viviam, fazendo uso tradicional do cerrado ^15^ .

Por outro lado, o Semiárido, área de ocupação antiga do território brasileiro e com baixa inserção no circuito econômico, é uma região marcada pela seca, com impactos no desenvolvimento econômico, social e ambiental que se reflete em piores indicadores sociais e de saúde. Embora seja um evento climático, seu impacto depende das atividades humanas, da vulnerabilidade social e das respostas das políticas públicas ^16^ .

No vetor Centro-Oeste, o meio técnico-científico informacional se estabeleceu sobre um território de herança técnica rarefeita, que absorveu o novo por meio da moderna produção agrícola associada à pecuária com uso corporativo do território. O aspecto não apenas tecnológico, mas ideológico que o sustenta pode ser descrito na agricultura globalizada, combinada à participação do Estado via financiamento. É, sem dúvida, o *cluster* que mais se integra ao circuito econômico global, por meio do agronegócio ^6^ , agregando 84 MRR, incluindo os de Rondônia, que se integram funcionalmente nesse vetor, em decorrência da expansão da cultura da soja e da agropecuária.

A Região Norte é caracterizada por rarefações demográficas e baixa densidade técnica, herdadas de períodos pretéritos do processo de ocupação espacial brasileira. A vastidão do território influencia a configuração de pontos de interligação; os pontos fluviais foram e são centrais. É um território de bioma florestal com uma vasta complexidade de relações ecológicas e sociais ^17^ , tem sua história marcada por surtos de intervenção externos e exploração predatória dos recursos naturais. O Sul e o Leste da região são zonas de tensão pela disputa de terras com o agronegócio, nas quais a expansão do capitalismo se processou simultaneamente à criação de empresas sob o incentivo e a direção do Estado, com marcada ênfase na matriz de transporte rodoviário ^18^ .

A partir dessas lógicas distintas foram delimitados dois *clusters:* Norte Águas e Norte Estradas. O primeiro aglutina os MRR marcados pela dinâmica dos rios, o segundo os pautados pelas rodovias. Em sete municípios observou-se uma lógica mista, antigamente suas dinâmicas eram pautadas pelos rios, mas recentemente ocorreu a chegada de rodovias, por causa dessa mudança optou-se por considerá-los como integrantes do *cluster* “Norte Estradas”.

No Norte Águas a complexidade da dinâmica dos rios impacta toda a vida da população. O rio é o meio de acesso à água, ao transporte, ao lazer ou a quaisquer tipos de serviços. Na época de vazante do rio, algumas comunidades ficam totalmente isoladas, devido à dificuldade de acesso por água ^19^ . Por outro lado, no Norte Estradas, os principais marcos são as rodovias, a exemplo da Transamazônica e Santarém-Cuiabá. Se por um lado, os MRR têm um percentual de indígenas quase 10 vezes superior ao conjunto da população brasileira, nesses dois *clusters* os percentuais são ainda mais elevados chegando a mais de 9% no Norte Águas ( Tabela 2 ).

**Tabela 2:** Indicadores de saúde e demográficos selecionados. Municípios rurais remotos e *clusters* , Brasil.

Indicador	Brasil	MRR	Clusters					
			Vetor Centro-Oeste	Norte de Minas	Matopiba	Norte Estrada	Norte Água	Semiárido
Consultas hab./ano ^a^	1,79	1,65	2,85	3,07	1,20	0,87	0,95	2,16
Visitas domiciliares de ACS hab./ano ^e^	1,57	2,58	2,67	2,93	2,31	2,19	2,81	2,70
Cobertura da ESF (%) ^g^	63,7	85,78	83,50	100	98,04	73,76	78,19	91,35
Internações SUS (100 hab./ano) ^a^	5,88	5,58	6,14	6,27	6,27	5,72	4,45	5,00
Internações SUS de alta complexidade (1.000 hab./ano) ^a^	4,4	1,3	2,0	3,1	1,4	0,7	0,6	1,6
Vidas cobertas por planos privados de saúde (%) ^c^	22,4	1,50	3,97	0,86	1,16	0,85	0,33	0,72
Coeficiente de mortalidade infantil ^a^	12,39	17,56	7,10	16,95	16,56	20,79	17,00	16,55
Óbitos por causas mal definidas (%) ^a^	6,06	10,94	8,93	15,23	9,25	13,15	13,92	9,37
Internações por condições sensíveis a atenção primária à saúde (%) ^d^	30,6	41,14	33,19	38,28	42,77	42,98	41,27	48,36
Nascidos vivos com pré-natal adequado (%) ^a,b^	70,76	52,81	66,82	71,97	58,9	45,72	39,85	62,3
Casos de câncer com início tratamento após 60 dias no SUS (%) ^a^	17,7	21,7	18,3	27,4	21,8	24,5	23,2	27
Indígenas na população (%) ^f^	0,43	4,62	4,42	4,14	1,99	6,17	9,17	0,22

ACS: agente comunitário de saúde; ESF: Estratégia Saúde da Família; MRR: municípios rurais remotos; SUS: Sistema Único de Saúde.

^a^ Calculado a partir de dados disponíveis no Datasus, ano referência 2019.

^b^ Início do pré-natal no primeiro trimestre e pelo menos seis consultas de pré-natal.

^c^ Calculado a partir de dados disponíveis na Agência Nacional de Saúde, referência junho de 2019.

^d^ Dados disponíveis no Datasus, ano referência 2015.

^e^ Calculado a partir de dados disponíveis no Sistema de Informação em Saúde para a Atenção Básica, ano de referência 2019.

^f^ Dados do Censo 2010.

^g^ Calculado a partir de dados disponíveis no e-Gestor Atenção Básica, com referência de junho de 2019.

A maioria dos MRR tem seus Índice de Desenvolvimento Humano Municipal (IDHM) classificados como baixos, apenas 13 estão na faixa mais alta, dos quais 12 estão no vetor Centro-Oeste. Por outro lado, o Norte Águas tem a maior concentração de municípios com IDHM na faixa “Muito Baixo”. As economias frágeis dos MRR podem ser evidenciadas pelo importante peso da administração pública nas econômicas locais, sendo que 81,2% tem o serviço público como a principal atividade que adiciona valor à economia. As exceções se localizam fundamentalmente no vetor Centro-Oeste e em Matopiba. No vetor Centro-Oeste destacam-se municípios que têm a agricultura como principal atividade e a pecuária como atividade secundária e, em Matopiba, a agricultura é a principal ocupação.

Observam-se diferenças entre os *clusters* no que se refere ao número de habitantes, área, densidade populacional, PIB *per capita* e percentual da população beneficiária do Bolsa Família ( Tabela 1 ). A análise do PIB *per capita* evidencia claramente a importante inserção no circuito econômico dos MRR do vetor Centro-Oeste e em menor grau dos municípios rurais remotos de Matopiba, enquanto a cobertura do Bolsa Família segue padrão inverso.

Outro ponto central é a distância dos MRR dos municípios classificados em hierarquias urbanas superiores. Esse dado pode ser entendido como uma medida indireta da dificuldade que a população enfrenta para chegar aos centros urbanos, nos quais são frequentemente mais ofertados os procedimentos e consultas ambulatoriais de média e alta complexidade e internações. Na Figura 2 podem ser visualizados os tempos em minutos para vencer as distâncias, dos seis *clusters* até a metrópole, capital regional, ou a um centro sub-regional. Em todos os casos observam-se diferenças (p < 0,01), indicando a existência de realidades remotas e não apenas um único remoto.

**Figura 2:** Tempo de deslocamento entre os municípios rurais remotos e as metrópoles, capitais regionais e centro sub-regional.

A. Kruskal-Wallis p < 0,01. Pós-hoc comparações múltiplas 1 ≠ 2, 3, 5, 6; 2 ≠ 3, 4, 5; 3 ≠ 4, 5, 6; 4 ≠ 5, 6; 5 ≠ 6.

B. Kruskal-Wallis p < 0,01. Pós-hoc comparações múltiplas 1 ≠ 2, 3, 5, 6; 2 ≠ 3, 4, 5; 3 ≠ 5; 4 ≠ 5; 5 ≠ 6.

C. Kruskal-Wallis p < 0,01. Pós-hoc comparações múltiplas 1 ≠ 2, 5, 6; 2 ≠ 4, 5; 3 ≠ 4, 5, 6; 4 ≠ 5, 6; 5 ≠ 6.

O Norte Águas apresenta as maiores distâncias entre seus municípios e os centros urbanos hierarquicamente superiores, com média de 4.565 minutos até a metrópole. Chama atenção que para 14 MRR, a ligação se dá diretamente com as metrópoles, especialmente Manaus, sem rede urbana intermediária e em 10 diretamente com as capitais regionais.

Os municípios do Norte Estradas também enfrentam grandes distâncias, um pouco menos da metade dos municípios (13) se liga diretamente às capitais regionais e às metrópoles Manaus e Belém. Os centros sub-regionais espelham sobremaneira o processo de ocupação e crescimento desses municípios, assentado via rodoviária, como Altamira, palco de conflitos ambientais e de ocupação de terra durante a construção da hidrelétrica de Belo Monte.

Com distâncias ainda na casa de mil minutos para a metrópole e de 500 para as capitais regionais, estão os MRR do vetor Centro-Oeste. Se esses tempos parecem pequenos comparados ao tempo dos *clusters* citados anteriormente, ainda são muito relevantes. Apenas oito dos MRR desse *cluster* se relacionam diretamente com a metrópole ou capital regional, enquanto 62 estabelecem relação com centros de zona e de sub-região, indicando a presença de uma rede de cidades bastante distribuída.

Os MRR de Matopiba apresentam médias de distância percorridas em cerca de 750, 290, 200 minutos respectivamente para a metrópole, capital regional e centro sub-regional. Da mesma forma que no vetor Centro-Oeste, observa-se uma ampla conexão com as cidades classificadas como centros de zona e de sub-região.

Em direção oposta, com distâncias e tempos bem menores, com múltiplas interligações com diversas cidades hierarquicamente superiores, estão o Norte de Minas e o Semiárido. No primeiro, as capitais regionais distam em média 215 minutos. No segundo, há um maior número tanto de capitais regionais, quanto de metrópoles, o que confirma a antiguidade dos processos de ocupação do território.

Na Tabela 2 é possível observar diferenças importantes nos indicadores selecionados de saúde e de desempenho de serviços de saúde, tanto entre o conjunto dos MRR e o restante do país quanto entre os *clusters.* Quanto à oferta e organização dos serviços de saúde, há predominância no modelo da Estratégia de Saúde da Família, com coberturas populacionais e número de visitas de agente comunitário de saúde superiores à média nacional. Apesar da alta cobertura, a questão da qualidade é um problema como demonstra o percentual de nascidos vivos com pré-natal adequado menor em todos os *clusters,* comparando ao quadro nacional, exceto no Norte de Minas Gerais e no comportamento das Internações por Condições Sensíveis a Atenção Primária em Saúde (ICSAPS). Uma APS forte e robusta demanda uma articulação com os demais níveis do sistema, as dificuldades para essa articulação ficam evidentes nos indicadores de internações de alta complexidade e percentual de casos de câncer com início do tratamento em menos de 60 dias após o resultado do anatomopatológico. Expressando a pequena capacidade econômica da população, a cobertura de planos privados é praticamente inexistente ( Tabela 2 ).

## DISCUSSÃO

A ausência de referenciais teóricos é uma lacuna importante nos estudos sobre a saúde nos cenários rurais e remotos ^20^ , nesse sentindo a incorporação do referencial teórico de Santos e Silveira ^6^ permitiu uma melhor aproximação dos diversos cenários rurais e remotos brasileiros. Os resultados encontrados indicam a existência de distintas realidades que, da perspectiva das políticas de saúde, podem e devem ser consideradas.

Internacionalmente, as políticas voltadas para garantia do acesso à saúde em áreas rurais têm apontado para a necessidade de: um modelo de atenção à saúde baseado em uma APS robusta, com forte componente comunitário e cultural; iniciativas amplas de telessaúde; reforço da capacidade logística de transporte de usuários e insumos, com lógicas distintas para atenção especializada, internações e situações de urgências; e a introdução de fortes mecanismos de fixação de profissionais nessas regiões ^21 , 22^ .

No caso brasileiro, é imperativo a formulação de políticas que dialoguem com as particularidades dos cenários identificados e revertam a situação observada nos indicadores de saúde apresentados. Tomemos por referência para essa discussão dois temas centrais para as políticas de saúde brasileiras: a construção das regiões de saúde e o modelo de atenção.

Uma das primeiras questões a se enfrentar é qual a capacidade de resposta regional *vis a vis* às diferentes inserções na rede de cidades. A inexistência de uma rede de cidades próximas, ou seja, ligações desses MRR com longínquas capitais regionais ou metrópoles, sem dúvida impacta negativamente nos processos de construção de regiões de saúde. Sem dúvida, esse desenho é mais factível nos MRR localizados a distâncias menores das capitais regionais e dos subcentros, como é o caso daqueles do Semiárido e do Norte de Minas, onde o investimento logístico em sistemas de transporte rodoviários dos usuários é mandatório e experiências como policlínicas regionais podem suprir as deficiências no acesso à atenção especializada, desde que garantido o transporte sanitário ^23^ . No entanto, essa mesma estratégia passa a ser de difícil operacionalização naqueles MRR em que a população precisa enfrentar imensos deslocamentos até os centros urbanos hierarquicamente superiores, como é o caso dos *clusters* de Norte Água e Norte Estrada, onde, sem dúvida, a oferta local, mais próxima de serviços especializados, mesmo sem economia de escala, é central para garantir acesso para os cidadãos que lá residem. Essas necessidades precisam se refletir no financiamento, especialmente das esferas estadual e nacional, pois a maior parte dos MRR tem economias bastante frágeis, incapazes de arcar isoladamente com os altos custos decorrentes dessas estratégias.

A ausência de transporte é um dos fatores mais relatados como barreira para que residentes de áreas rurais acessem serviços de saúde de maior complexidade. Muitas vezes os custos para realizar o deslocamento são assumidos de forma direta pelos usuários, comprometendo o parco orçamento familiar e configurando um gasto catastrófico ^24^ . Esse serviço deve ser implantado, mantido e custeado pelos três entes federados, de forma solidária. No Brasil, existe, desde 1999, a previsão de pagamento para uma parcela desses deslocamentos por meio do “Tratamento Fora de Domicílio”, no entanto são inúmeras as fragilidades dessa política ^25^ .

A garantia de atendimento de urgência é um grande desafio em cenários como esses e recoloca a discussão do papel dos hospitais de pequeno porte e unidades mistas. Se por um lado, a literatura aponta constrangimentos à oferta de uma atenção de qualidade, por outro, a garantia de uma primeira atenção de emergência é mandatória, dado os longos tempos de deslocamento e distâncias envolvidos ^24^ . É mandatória a ampliação do escopo de práticas dos profissionais de saúde, com suporte de telessaúde, associada a política de financiamento específica.

Quanto ao modelo de atenção, os dados indicam uma expressiva presença da Estratégia Saúde da Família nesses municípios, o que é uma grande potencialidade. A literatura é profícua em demonstrar que modelos de atenção à saúde baseados na APS são associados a melhor desempenho do sistema de saúde de uma forma geral. Uma APS forte teria condições de diminuir as diferenças no acesso e nos resultados em saúde entre as populações rurais e urbanas. Mas, os desafios enfrentados nas localidades rurais são ainda maiores, cobrando novos desenhos de oferta de serviços, uma competência clínica ampliada, forte componente comunitário e cultural, além da ênfase em ações de promoção, prevenção e participação social ^26^ . Um outro ponto positivo no modelo predominante é a presença dos agentes comunitários de saúde, que muitas vezes são a única garantia de ligação de parcela da população aos serviços de saúde, entretanto, não existe preparo e nem um acompanhamento previsto para que possam atuar com maior competência diante dessa realidade, o que deveria ser revisto.

Os tipos mais comuns de oferta de serviços de APS para essas regiões são os modelos de visita, no qual as equipes se deslocam da sede e vão às localidades mais remotas esporadicamente, mesmo que a intervalos regulares, o que dificulta tanto o tratamento oportuno como a continuidade do cuidado. Essa estratégia se baseia na premissa da impossibilidade de fixar profissionais de saúde, especialmente médicos e enfermeiros. Sem dúvida, políticas para fixação desses profissionais são mandatórias e as mais bem-sucedidas se articularam com os processos formativos desde a graduação. No Brasil, o Programa Mais Médicos foi uma importante iniciativa para a reversão desses vazios assistenciais ^27^ . Outro ponto positivo a ser destacado na política de atenção básica brasileira foi a incorporação da dimensão cultural, bem expressa na Política Nacional de Saúde Integral das Populações do Campo e da Floresta.

No entanto, apesar desses aspectos positivos, muito ainda precisa ser realizado. Uma iniciativa central para a melhoria do acesso à saúde é o uso de tecnologia da informação. As mais variadas iniciativas de telessaúde têm sido reportadas como importantes alternativas nos mais diversos cenários rurais ou remotos mundialmente ^28^ . Os resultados aqui encontrados, expressos nas imensas distâncias entre os municípios e os centros urbanos especialmente nos *clusters* do Norte Água, Norte Estrada e vetor Centro-Oeste, sugerem que as tecnologias de informação são um investimento essencial para garantir a diminuição das iniquidades do acesso à saúde. Ressalta-se que é muito ampla a gama de possibilidades encontrada nas experiências internacionais, especialmente nos países de alta renda que enfrentam essa realidade, indo muito além do que vem sendo proposto no telessaúde brasileiro, com inclusão até mesmo de exames, procedimentos e atendimentos de urgência, que são realizados em conjunto entre as equipes locais e as remotas ^29^ .

## CONCLUSÕES

Este estudo mostra que os municípios rurais remotos brasileiros não são homogêneos e que as distintas características socioespaciais e de inserção na lógica econômica demandam políticas de saúde customizadas para as diferentes realidades. Políticas de financiamento mais solidárias, adequação dos desenhos das redes regionais de saúde, políticas específicas para provimento de recursos humanos, além de um incentivo a uma APS robusta, com escopo de práticas ampliada e com forte componente cultural e comunitário devem ser consideradas como políticas prioritárias pelos gestores. A compreensão dessas especificidades e a elaboração de políticas específicas para esses territórios são mandatórias para garantir o direito à saúde para os cidadãos que aí residem, com equidade e integralidade, contribuindo para tornar visíveis essa parcela muitas vezes invisível da população brasileira.
